# Exploring the relationship between peer attachment, and disordered eating behaviours and body dissatisfaction in adolescence: a systematic review

**DOI:** 10.1186/s40337-025-01273-3

**Published:** 2025-07-08

**Authors:** Clarice Chan, Cecily Donnelly, Aphrodite Eshetu, Dasha Nicholls

**Affiliations:** 1 Faculty of Medicine, Imperial College London, London, UK; 2 Division of Psychiatry, Department of Brain Science, Faculty of Medicine, Imperial College London, 2nd Floor Commonwealth Building, Du Cane Road, London, W12 0 NN UK

**Keywords:** Eating disorders, Attachment, Peers, Adolescents, Body dissatisfaction, Disordered eating

## Abstract

**Objective:**

Adolescence is a critical period in the development of eating disorders (ED) and the influence of peers becomes increasingly evident in shaping behaviour. Insecure attachment to parents is associated with higher ED risk, but the impact of attachment to peers is unknown. This systematic review aims to ascertain the role of peer attachment in ED symptoms and body dissatisfaction in adolescents.

**Methods:**

Electronic databases Medline, Embase, PsychINFO, and Scopus were searched using the search terms related to adolescents, eating disorders, peers, and attachment.

**Results:**

Out of the 19 included studies (n = 15 cross-sectional, n = 4 Longitudinal), most (n = 17) used the Peer subscale of the ‘Inventory of Parent and Peer Attachment (IPPA)’ to measure peer attachment. The prevalence of insecure attachment was higher in populations with ED symptoms compared to those without. Lower attachment scores were associated with higher ED symptomology and body dissatisfaction. Out of the 3 subscales of the IPPA, alienation emerged as the most significant predictor of symptomology.

**Discussion:**

Insecure attachment to peers may be a risk factor in the development of ED symptoms and body dissatisfaction, but the protective role of secure attachment remains unclear. More longitudinal research is required to disentangle this relationship and ascertain the clinical significance of peer attachment in prognosis or intervention.

## Introduction

With the prevalence of eating disorders (ED) continuing to rise, there has been a corresponding expansion of research focused on understanding factors influencing the development and outcome of disordered eating behaviours. Despite progress in treatment, global probable ED prevalence rose 7.8% from 2013 to 2018 [1]. Additionally, the isolating repercussions from the COVID-19 pandemic further increased this burden, with increased isolation, exposure to anxiety-provoking media and exacerbated stress [2, 3]. Further concern arises from an estimated 41.9 million unrepresented eating disorders and the hidden issue of disordered eating behaviours that do not reach diagnostic threshold [4–6]. Practices such as binge-eating and dietary restriction can increase the risk of developing an eating disorder, as well as poor perceived health and psychological distress [7, 8]. Increasing attention is turning public health approaches to ED, with a growing emphasis on identifying early risk and protective factors to inform prevention strategies.

Adolescence arguably holds the greatest risk in terms of body image and eating attitudes because of the inherent biopsychosocial changes in this developmental period [9–11]. The influence of familial relationships during this critical period are well established, and factors such as familial support, teasing, and mealtime environment have been implicated in disordered eating[12, 13]. Less attention has been given to peer relationships, which begin to play an increasingly larger role in most adolescents’ lives. As young people become more conscious of societal standards and receptive to the opinions of others, one of the greatest developmental challenges in this adjustment period is the formation and changing nature of new relationships [14, 15]. Adolescents become progressively more amenable to influence from peers: friends can promote healthy behaviours and emotional coping but may also encourage dangerous behaviours such as smoking and delinquency [16–19].

With respect to ED risk, factors such as self-image derive in part from the feedback we have received from others [20, 21]. Similarly, quality of friendship can determine ideas such as value and self-worth [22, 23]. Further influence derives from the increasing use of social media with its volumes of body-image related content [24]. A protective aspect of peer support derives from actions such as discouraging unhealthy behaviours or helping dismantle unrealistic body ideals or beliefs [25–27]. Support from peers has also been demonstrated to be a facilitator in recovery from anorexia nervosa [28, 29].

However, influence of friends may also be detrimental to body image and eating behaviours. Encouragement of unhealthy eating behaviours can derive directly from teasing, bullying or pressure from others to lose weight [25, 30–32]. It may also arise indirectly through social comparison or a perceived normative pressure for certain body types [15, 16, 21, 25]. Studies suggest that modelling and imitation can lead to perpetuation of these behaviours [21, 30].

Peer relationships can be complex. Studies suggest that the strength of friendship, for example a best friend compared to a casual friend, exerts differing impacts on eating and weight-related behaviours [16, 33]. Friends with a stronger bond have greater potential to internalise each other’s thoughts [34]. Yet, the specific impact of more intimate friendships, rather than the social context, has yet to be explored in detail.

Attachment theory is a well-established psychological framework that describes the relationship between infants and their parents [35–38]. It incorporates many concepts, mainly that infants will seek proximity to a security figure, and that the nature of this relationship will inform their internal working model. Experiences in early childhood are therefore reflected in how a person perceives themselves and their later behaviours. Internalised attitudes and beliefs around body weight and eating contribute to self-representation [39–41]. Body image is enhanced by the feelings of rejection that are associated with attachment insecurity [42, 43].

Although the origins of attachment theory lie in parent–child relationships, the increasing dependency on peers for intimacy and support during adolescence has seen the concept of attachment increasingly applied to peers, such as the safe-haven function, intimacy, and security [44–47]. This is especially evident in children with insecure attachment to their guardians [48]. Observing peer attachment patterns has many implications in psychology, such as behavioural issues and emotion regulation difficulties [49, 50]. Those with secure peer attachment often display prosocial behaviour that is carried forward into adulthood [17, 49, 51, 52].

A relationship between insecure attachment and eating pathology has been demonstrated in adults. For example, Faber et al. found that lower attachment security both distinguishes those with eating pathology from the general population and characterises disordered eating behaviours within the general population [53]. Lev-Ari, Baumgarten-Katz and Zohar found that women with anxious attachment styles were more likely to report dissatisfaction with their body [54]. Recent studies have begun to replicate these findings in adolescents. Jewell et al. reviewed 22 studies and summarised the connections between attachment, mentalisation and eating disorders [55]. They concluded that attachment insecurity was associated with greater eating pathology in adolescence, supported by all included studies, bar one, exploring this concept.

## Objectives

Non-familial attachment patterns are under-studied [40]. Thus, this systematic review aims to determine the role of attachment to peers in the development of ED symptoms and body dissatisfaction.

### Methods

The methodology for this systematic review was developed according to PRISMA 2020 guideline recommendations [56]. Articles were sourced from electronic databases MEDLINE, EMBASE, PsychInfo, and Scopus in April 2024. There were no limits to date of publication. The search was based on four constructs: adolescents, eating disorders, peers, and attachment. Example search terms include ‘adolescen*’, ‘disordered eating’, ‘eating disorder*’, ‘peer*’, and ‘attach*’. A wide range of search terms were used to ensure all relevant papers were included, for example the use of ‘obesity’ and ‘overweight’ to encompass ED that are seen more frequently in individuals at higher weight [57, 58]. Additionally, the names of established measures of peer attachment were included, as reported by Jewell et al. [59]. There was an additional screen of references. A full search strategy can be found in Appendix 1.

### Eligibility criteria

The inclusion criteria were as follows:

- reported an outcome of ED, ED symptoms, or body image/satisfaction
- adolescents between the ages of 10 and 19, as per WHO definition of adolescence [60] from clinical or non-clinical populations
- included a standardised measure of attachment to peers as the predictor
- used any study design

Studies that observed participants outside age criteria were included if the majority of participants lay within this range. Authors were contacted if this information was not available. For longitudinal studies, the measurements of peer attachment and eating behaviours must have been undertaken when the participants were 10–19 years old.

Exclusion criteria were:

- observed individuals over the age of 19 years old, or under 10 years old
- measured a general attachment style that encompasses the relationship with parents as well as peers
- systematic reviews or meta-analyses

### Study selection and risk of bias assessment

**Fig. 1:**
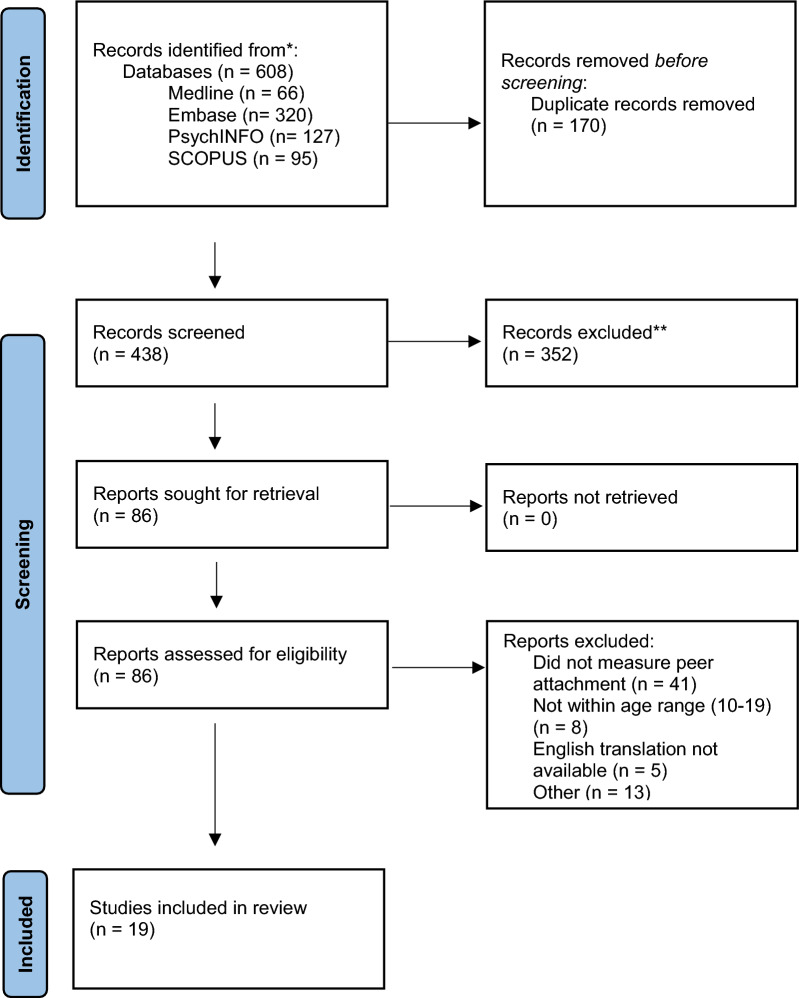
PRISMA flowchart displaying the study selection process. A total of 19 studies met criteria for inclusion

The study selection process is presented in the PRISMA flowchart (Fig. 1). Selection was completed using Covidence and screening was completed by two independent researchers, CC and AE. 438 studies were identified from the initial search and 352 were excluded in the title and abstract screen. 86 articles were selected for full-text review. 19 studies met the criteria for inclusion and were included in the final review [61–79]. Due to heterogeneity between outcomes, a meta-analysis was not possible and therefore a narrative synthesis is provided, with results grouped based on similarity of outcomes.

**Fig. 2:**
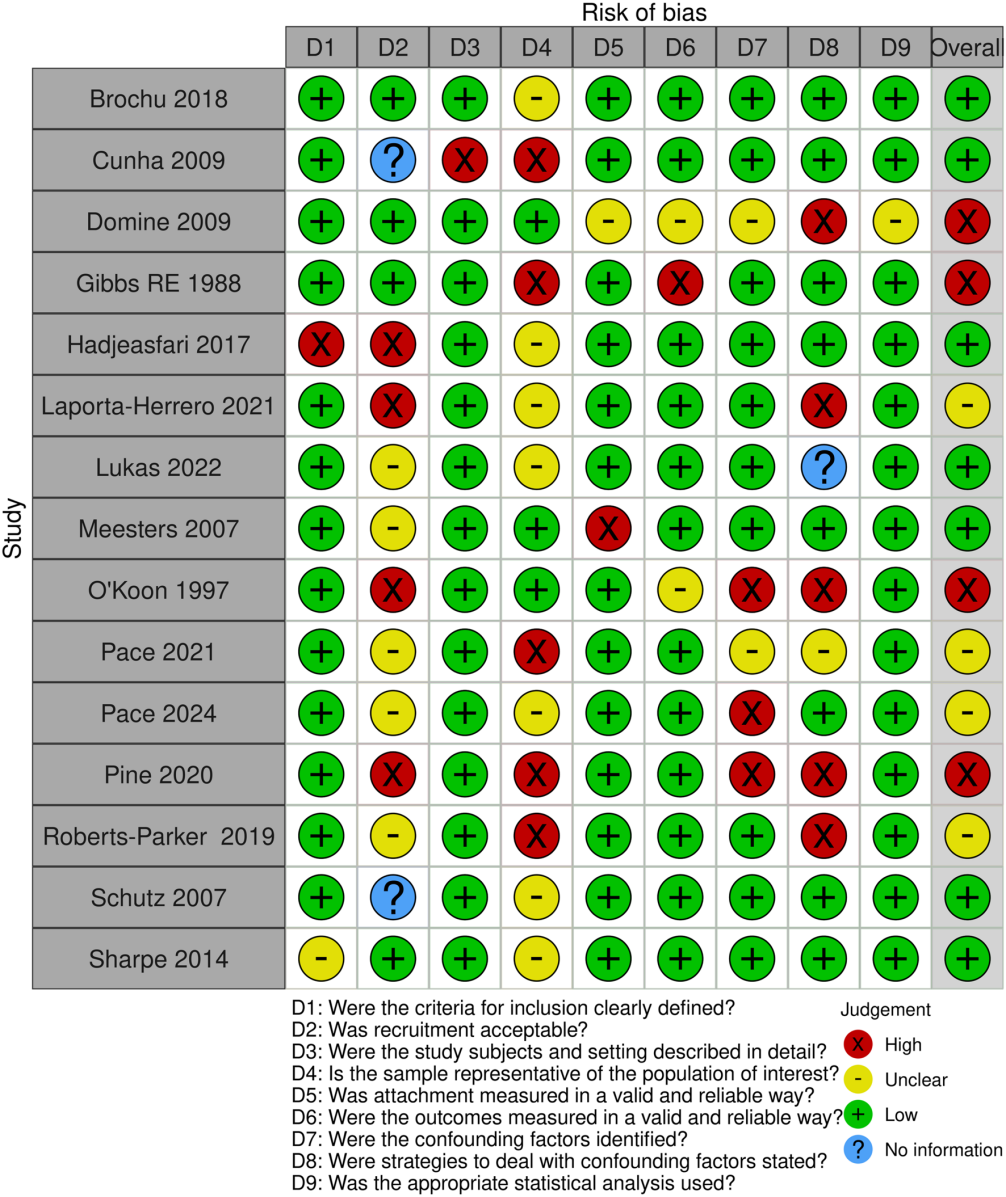
Risk of Bias assessment (Cross-sectional studies). Results of risk of bias assessment using an adapted version of The Joanna Briggs Institute (JBI) Critical Appraisal Checklist for analytical cross-sectional study and additional question from the McMaster University risk of bias tool for cross-sectional studies [80, 81]. Tables were generated using the ROBIVS tool [130]

**Fig. 3:**
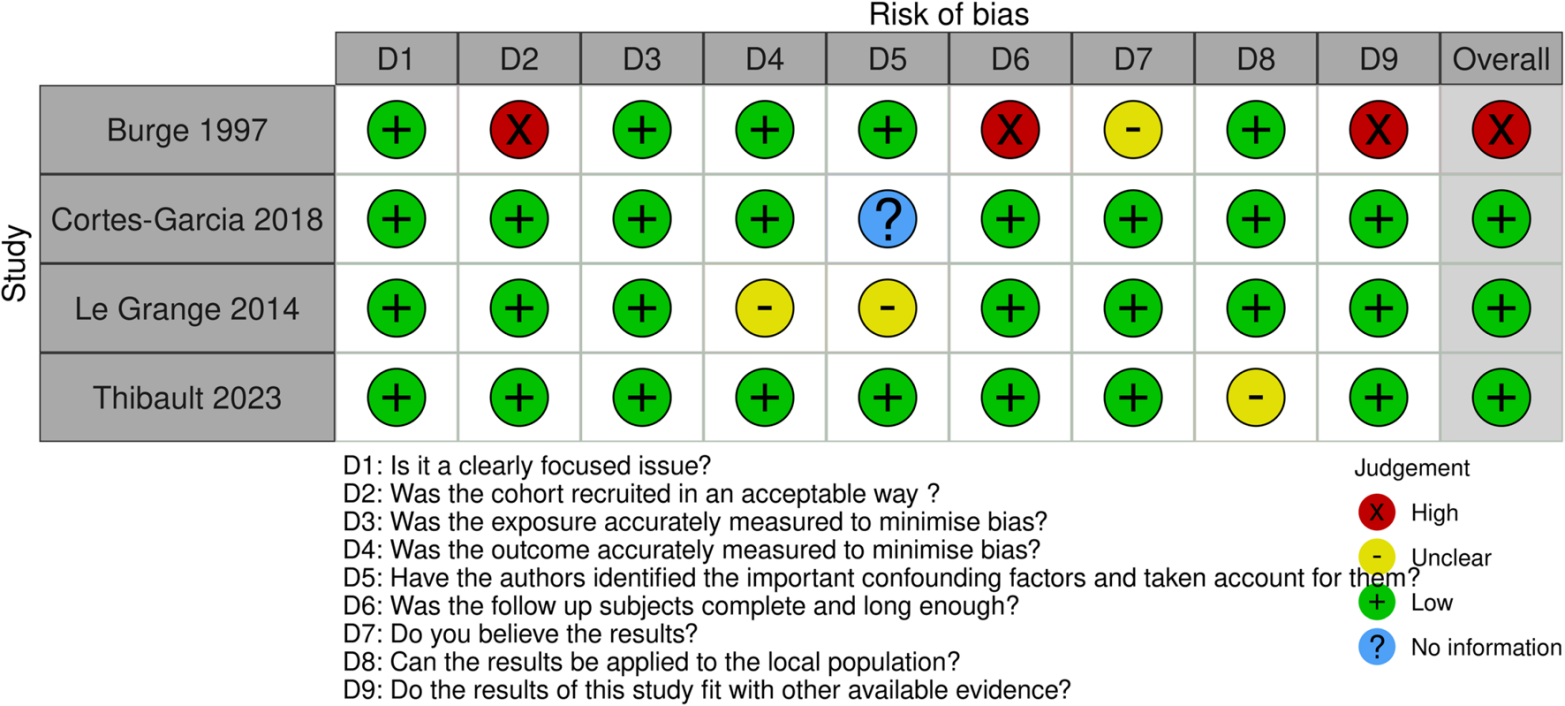
Risk of Bias assessment (Longitudinal). Results of risk of bias assessment using an adapted version of the CASP checklist for cohort studies [82]. Tables were generated using the ROBIVS tool[130]

Risk of bias assessment was conducted using an adapted version of The Joanna Briggs Institute (JBI) Critical Appraisal Checklist for analytical cross-sectional study with an additional question from the McMaster University risk of bias tool for cross-sectional studies [80, 81] (Fig. 2). Longitudinal studies were assessed based on an adapted version of the CASP checklist for cohort studies [82] (Fig. 3). Particular areas of interest were the recruitment methodology, identification and accounting of confounders, and generalisability to the wider population. Additional measures of whether the sample was representative of the population of interest were included. Data extraction was completed using Microsoft excel.

## Results

Nineteen studies met the inclusion criteria, and their characteristics and main findings are displayed in Tables 1 and 2. Publishing date ranged from 1988 to 2024. Fifteen studies included cross-sectional designs. Ten observed single-sex populations. Sample size ranged from 39 [73] to 3890 [63].

**Table 1:** Characteristics of included studies

First author and year	Design (country)	Sample size	Age (mean)	Sex	Attachment measure	Outcome(s) of interest
Brochu 2018[61]	Cross-sectional (Canada)	186	12–18 (15.36)	All female	IPPA	Eating disorder symptom severity: EDRC composite of the Eating Disorder Inventory (EDI-3)
Cunha 2009[62]	Cross-sectional (Portugal)	68	13–23 (clinical:17.26 control:17.18)	All female	IPPA	Prior clinical diagnosis of anorexia nervosa by a psychiatrist according to DSM IV Risk of eating disorder: EDI-2
Domine 2009[63]	Cross-sectional (Switzerland)	3890	16–20	All male	Modified IPPA	Concerning thoughts and behaviours indicative of disordered eating via Weight and Eating Concerns Inventory (WECI) with two added study derived items
Gibbs 1988[64]	Cross-sectional (US)	727	14–20 (16.88)	All female	IPPA	Classification as (1) anorexic (2) anorexic-like (3) bulimic or (4) ‘normal’, according to modified DSM-III criteria
HadjeasGari 2017 [65]	Cross-sectional (US)	42	12–17 (14.71)	All male	IPPA	Eating disorder risk via Eating disorder examination questionnaire (EDE-Q)
Laporta-Herrero 2021[66]	Cross-sectional (Spain)	260	13–17 (15.35)	70% female 30% male	IPPA	Diagnosis with an eating disorder (diagnostic criteria ICD-10) Body dissatisfaction via Body Shape Questionnaire (BSQ-34) (Spanish version)
Lukas 2022[67]	Cross-sectional (Germany)	75	12–18	All female	IPPA	Eating disorder symptoms measured with the Eating Disorder Inventory 2 – EDI
Meesters 2007[68]	Cross-sectional (The Netherlands)	405	10–16 (12.5)	224 female 181 male	Relationship Questionnaire for Children (RQ-C)	Modified Children’s Eating Attitudes Test (ChEAT) score: food preoccupation and dieting and muscle preoccupation subscales
O’Koon 1997[69]	Cross-sectional	167	16–18 (17)	95 female 72 male	IPPA	Body Image via offer self-image questionnaire (OSIQ)
Pace 2021[70]	Cross-sectional (Italy)	112	14–18	All female	Family and Friends Interview (FFI), peer relations scale	BE behaviours via the Binge Eating Scale (BES)
Pace 2024[71]	Cross-sectional (Italy)	423	12–18 (16.88)	67.8% female 32.2% male	IPPA	BE behaviours via the Binge Eating Scale (BES)
Pine 2020[72]	Cross-sectional (US)	136	12–17 (14.39)	56% female 44% male	IPPA-revised	Emotional eating via emotional eating scale for children, Loss-of-control eating via the eating disorder examination interview
Roberts-Parker 2019[73]	Cross-sectional (US)	39 (pre-test)	12–14	All female	IPPA-Revised	High or low risk for an eating disorder as determined by the Children’s Eating Attitudes Test (ChEAT)
Schutz 2007[74]	Cross-sectional (Australia)	327	15–16 (15.9)	All female	IPPA	Body attitudes questionnaire, feeling fat subscale Extreme Dutch Eating Behaviour Questionnaire Restraint Subscale (DEBQ-R) Extreme Weight Loss Behaviours scale (EWLB) Eating Disorder Inventory Bulimia subscale (EDI-B)
Sharpe 2014[75]	Cross-sectional (UK)	216	13–16 (13.57)	All female	IPPA	Eating Disorder Examination Questionnaire (EDE-Q) Body Esteem Scale for adults and adolescents (BES)
Burge 1997[76]	Longitudinal (US)	155	(18.17)	All female	IPPA	Eating Disorder symptomology: structured interview
Cortés-García 2019[77]	Longitudinal (Spain)	904	10–16 (10.83)	49.4% girls 50.6% boys (T1)	IPPA-Revised translated into Spanish	Children’s Eating Attitudes Test (ChEAT)
Le Grange 2014[78]	Longitudinal prospective (Australia)	1300	15–16	51.3% girls 48.7% boys	IPPA (modified)	Abnormal eating attitudes and behaviours via the Drive for Thinness and Bulimia subscales (EDI), body dissatisfaction subscale and two study-derived questions
Thibault 2023[79]	Longitudinal (Canada)	143	12–17 (14.84)	All female	IPPA	Eating Disorder Inventory‑3 (EDI‑3)

**Table 2:** Main findings of included studies

First author and year	Main findings for total attachment	Main findings for trust	Main findings for communication	Main findings for alienation
Brochu 2018[61]		Negative correlation with eating disorder symptom severity (r = − 0.38, *p* < 0.001)	Negative correlation with eating disorder symptom severity (r = − 0.33, *p* < 0.001)	Positive correlation with eating disorder symptom severity (r = 0.50, *p* < 0.001) Overall relationship quality predicted 37% of symptom severity and peer alienation contributed 27% (*p* < 0.01) Alienation was associated with low self-esteem (b = 0.27 CI [0.18, 0.38]) and negative mood LSE (b = 0.19 CI [0.09,0.32]), which were both associated with ED symptom severity
Cunha 2009[62]	Total attachment scores were lower in those with anorexia nervosa (t = − 4.17, *p* < 0.0005) compared to the control	Trust scores were lower in those with anorexia nervosa (t = − 3.27, *p* < 0.0005) compared to the control	Communication scores were lower in those with anorexia nervosa(t = − 3.02, *p* < 0.005) compared to the control	Alienation was greater in those with anorexia nervosa (t = 6.20, *p* < 0.0005) compared to the controls Peer alienation was the primary distinguishing variable between the anorexia nervosa group and control (r = 0.71)
Domine 2009[63]	Peer attachment (relationship with peers) did not differ between the control group (no concerning eating behaviours) and those with concerning attitudes or behaviours			
Gibbs 1988[64]		No significant difference in trust between groups	No significant difference in communication between the groups	Peer alienation significantly higher in the groups that exhibited restrictive or purging eating behaviours (labelled “anorexic-like” and “bulimic”) compared to those who did not. (F [2,119) = 5.27, *p* < 0.01) Discriminant function coefficient of bulimia and normal groups r = − 0.20
Hadjeasgari2017[65]	Eating disorder risk decreased as peer attachment level increased (r = − 0.621, *p* < 0.001)			
Laporta-Herrero 2021[66]		No significant difference in trust between the eating disorder group vs the control group	Communication was lower in the eating disorder group vs the control group (F (1) = 4.53, *p* < 0.01)	Alienation was higher in the eating disorder (ED) group vs the control group (F(1) = 5.67, *p* < 0.01) Higher perceived alienation was associated with body dissatisfaction (r = 0.37, *p* < 0.05) in the ED group
Lukas 2022[67]	Adolescents with AN had lower total relationship quality scores (across trust, communication, and alienation) compared to the healthy control group, with medium to large effect sizes	Adolescents with AN reported lower levels of trust in their relationships with peers compared to the HC group, with medium to large effect sizes	Adolescents with AN reported lower levels of communication quality in their relationships with peers (friends) compared to the HC group, with medium to large effect sizes	Adolescents with AN reported higher levels of alienation in their relationships with peers (friends) compared to the HC group, with medium to large effect sizes
Meesters 2007[68]	No significant correlations between insecure attachment to peers and food preoccupation/dieting in girls and boys			
O’Koon 1997[69]	Positive correlation between peer attachment and positive body image (r = 0.24, *p* < 0.05) Peer attachment did not predict positive body image			
Pace 2021[70]	Neither frequency of contact nor quality of contact with a best friend differed between those with a high risk of binge eating and those not at risk of binge eating			
Pace 2024[71]	Negative correlation between total IPPA score and BES (= − 0.24) Higher attachment scores indirectly mediated by alexithymia (beta = − 0.06, SE = 0.03), were associated with reduced BES Girls: indirect (beta = − 0.01 ) correlation between high attachment scores and reduced BES Boys: Only direct correlation between high attachment scores and reduced BES ((beta = − 0.02)	Correlation between lower trust scores and BES (− 0.22)	Correlation between lower communication scores and BES (− 0.19)	Correlation between lower alienation scores and BES (− 0.28)
Pine 2020[72]	Peer attachment did not differ between those with ‘elevated anxiety’, ‘loss of control eating’ and ‘elevated anxiety and loss of control eating’ Indirect pathway through peer attachment was associated with greater depressive symptoms (r = − 0.12, SE = 0.05, *p* = 0.01), which was associated with emotional eating (r = 0.03, SE = 0.01, *p* < 0.01)			
Roberts-Parker 2019[73]	Negative correlation between peer attachment and eating disorder risk (r(39) = − 0.44, *p* = 0.006, effect size 0.19). Also found in the high eating disorder risk group (r(12) = − 0.81, *p* = 0.001). Non-significant correlation in the low-risk group			
Schutz 2007[74]		Negative correlation with bulimic symptoms (r = − 0.15, *p* < 0.01),	Non-significant correlations with outcomes	Body dissatisfaction: Positive correlation with alienation (r = 0.27, *p* < 0.001), alienation greater in the high-score group (*p* < 0.005, effect size 0.12) Dietary restraint: Positive correlation with alienation (r = 0.20, *p* < 0.001), alienation greater in the high-score group (*p* < 0.005, effect size 0.06) Extreme weight loss behaviours: Positive correlation with alienation (r = 0.21, *p* < 0.001) Bulimic symptoms: Positive correlation with alienation (r = 0.37, *p* < 0.001), alienation greater in the high-score group (*p* < 0.005, effect size 0.20)
Sharpe 2014[75]		Higher scores predict lower body dissatisfaction (R^2^ = 0.07, F = 14.24, *p* < 0.01) but not eating pathology	Higher scores predict lower body dissatisfaction (R^2^ = 0.03, F = 6.53, *p* < 0.01) and, when accounting for depression, eating pathology (β = 0.20, *p* < 0.005)	Higher scores predict greater eating pathology (R^2^ = 0.07, F = 16.92, *p* < 0.01) and body dissatisfaction (R2 = 0.09, 20.37, *p* < 0.01)
Burge 1997[76]		Higher scores predicted subsequent symptomology when in interaction with previous symptomology (R^2^ change = 0.03, *p* < 0.05)	Higher scores predicted subsequent symptomology when in interaction with previous symptomology (R^2^ change = 0.04, *p* < 0.01)	Positively correlated with symptomology at 12-month follow up (r = 0.18, *p* < 0.05) predicted subsequent symptomology when in interaction with previous symptomology (R^2^ change = 0.16, *p* < 0.01) and without interaction terms (R^2^ = 0.02 *p* < 0.05)
Cortés-García 2019[77]	Positive correlation with disordered eating in boys at time 1 (r = 0.14, p < 0.01) and time 4 (r = 0.14, p < 0.01) Cross-lagged panel analysis Girls: No significant correlations Boys: significant correlation between attachment to peers at time 1 and disordered eating at time 2 (r = 0.10, p < 0.05)			
Le Grange 2014[78]	Correlation between higher peer attachment and abnormal eating attitudes and behaviours (r = 0.20)			
Thibault 2023[79]		Correlation between lower trust scores and greater AN symptom severity at T2 (r = − 0.21, *p* = 0.01)	Correlation between lower communication scores and greater AN symptom severity at T2 (r = − 0.20, *p* = 0.02)	Correlation between higher alienation scores and AN symptom severity at T2 (r =.47, *p* = 0.00) Greater alienation with peers at T1 (b = 1.20, se =.53, *p* = 0.03) predicted greater AN symptom severity at T2, above and beyond the influence of adolescent girls’ general psychological maladjustment at T1, explaining an additional 2.4% of the variance

Most studies measured peer attachment using the self-report questionnaire ‘Inventory of Parent and Peer Attachment’ (IPPA) [83]. This widely used and validated questionnaire has three subscales measuring the amount of trust you feel towards friends (trust), quality of communication with friends (communication), and how isolated or rejected you feel (alienation). Trust and communication subscales are summed, while alienation is subtracted from the score. A low score indicates insecure attachment, and a high score suggests secure attachment. Outcomes included diagnosis of an ED, ED symptoms and body image dissatisfaction. The most utilised measure was the ‘Eating Disorder Inventory’ (EDI), a popular clinical tool used for screening and monitoring of ED [84, 85]. Most studies used the Eating Disorder Risk Composite (EDRC) score from the EDI. Four studies, all utilising the IPPA as their attachment measure, included an outcome measure of body image [66, 69, 74, 75].

Following quality assessment, five studies were at high risk of bias: Domine, Gibbs, O’Koon, Pine et al., and Burge et al. (see Figs. 2 and 3). Ten studies had low risk of bias and four were determined to be of unclear risk. Strengths included clearly focused questions and moderate sample sizes.

### Overall peer attachment: relationship with disordered eating/behaviours

Eleven studies explored the relationship between overall peer attachment and ED symptoms, with mixed results [62, 63, 65, 67, 68, 70–73, 77, 78]. One study investigated the relationship between overall attachment score and body dissatisfaction [69].

#### Cross-sectional

Cunha et al. and Lukas et al. both investigated the relationship between attachment quality and ED symptoms in adolescent females with AN compared to a healthy control group[62, 67]. Both found that patients with AN reported lower total attachment scores. Limitations lie in the small sample size, exclusion of participants from the control group if they were receiving treatment, recruitment of participants from a specific university department, and contacting patients from previous studies or those who had signed up to be contacted, raising questions about representativeness.

Domine et al. utilised data from a national survey database to assess the number of weight concerns and ED behaviours in school-aged boys [63]. The participants were arranged in groups according to whether they exhibited a concern, a behaviour, or neither. Items from the IPPA were used as a measure of relationships with peers. There were no significant differences in peer relationships between those who did not report any relevant concerns or behaviours and groups who did report a concerning thought or behaviour. A limitation is found in the extraction of only 4 items from a 25-item scale (IPPA). While this literature does not explicitly describe the recruitment procedure and sample, a referenced secondary document provided this information[86].

Hadejasgari, in a non-clinical male-only population recruited through study promotion to parents, found that greater peer attachment security, measured via IPPA, was associated with lower ED symptomology[65]. However, the small sample size and recruitment through advertisement to parents introduces bias.

Meesters et al. administered a modified ChEAT questionnaire to school students, finding non-significant correlations between attachment and ED symptomology[68]. This study used the Relationship Questionnaire for Children (RQ-C) which asks participants to select out of 4 statements which they most related to. While the original relationship questionnaire has good validity, the modified RQ-C has not been psychometrically evaluated[87].

112 girls attending high school were studied by Pace et al. The sample comprised of 56 girls whose Binge Eating Scale (BES) score classified them as high risk, and 56 age-matched controls with a low-risk BES score[70]. Utilising the semi-structured Family and Friends Interview, the authors were able to assess peer attachment using the peer domains of the interview and found no significant differences in peer attachment between those who were at risk for binge-eating and those who were not at risk. Pace et al. also compared relationships with siblings and found that those at risk for binge eating did not differ in sibling relationships compared to those who were not at risk. This study is limited by the restricted representation through its female-only population, which is due to no males consenting to inclusion.

Pace et al. conducted a cross-sectional study examining the role of alexithymia in the relationship between peer attachment and binge eating symptoms in 423 adolescents aged 12–18 years [71]. The study similarly utilised the IPPA to assess attachment and the BES. Findings indicated that good peer attachment was associated with reduced alexithymia, which in turn was linked to lower binge eating symptoms. Gender-specific analyses revealed differing pathways. In boys, while good attachment reduced alexithymia, this did not translate to a significant reduction in binge eating symptoms. In contrast, girls showed a clear pathway where good attachment reduced alexithymia, which then led to fewer binge eating symptoms. The study highlighted the importance of secure peer attachments in mitigating disordered eating behaviours, with alexithymia playing a mediating role.

Pine et al. studied adolescents who were dependent on military personnel, including those who were at a high risk of obesity or binge-eating [72]. Insecure attachment, measured via IPPA, was associated with greater depressive symptoms, which in turn was associated with emotional eating. There was no difference in peer attachment between participants who reported loss-of-control eating compared to those with elevated anxiety or both. Observing only high-risk military dependents greatly reduces generalisability. Additionally, in this high-risk-of-bias study, the loss-of-control eating group only made up 6% of the sample and separation into an ED and anxiety group, despite the well-documented association between anxiety and eating disorders, may reduce generalisability.[88].

Roberts-Parker assessed attachment to peers in non-clinical female adolescents in their thesis, and whether they had high or low ED risk, using the Children’s eating attitudes Test (ChEAT) [73]. Higher IPPA-R-peer scores were associated with a reduced ED Risk. Among the high-risk group, the same relationship was observed. However, the already small sample size (n = 39) was furthered narrowed in the second analysis, which omitted a group of participants who score “middle-risk”.

#### Longitudinal

Le Grange et al. obtained data from the Australian longitudinal data collection, observing IPPA scores [78]. In contrast with other studies, secure attachment was associated with greater abnormal eating attitudes and behaviours. There is strength in the large sample size; however, validity is limited by the shortening of the IPPA-peer from 25 items to 8. There was also attrition bias, with greatest loss from the low socio-economic status demographic. Information regarding data collection was sourced from a secondary document [89].

A cross-lagged panel analysis also observed comparable findings [40]. Cortés-García et al. measured peer attachment and disordered eating in a non-clinical sample at four time points. Positive associations were found in boys at time 1 (age 10) and time 4 (age 16). Higher peer attachment levels, signifying security, at time 1 were associated with higher reported disordered eating at time 2 (age 12) in boys. Although significant, the magnitude of the association was small and there was high attrition bias from the 50% drop in participants between times 3 and 4.

### Overall peer attachment: relationship with body dissatisfaction

O’Koon found a correlation between higher peer attachment and positive body image in adolescents from a mix of religious, public, and private schools[69]. Secure attachment, however, did not predict body confidence. Hand-selection of schools and students, along with differing selection methods between schools, contributes to high risk of bias. Additionally, O’Koon used a significantly shortened version of the ‘Offer self-image questionnaire’ (OSIQ).

### Peer attachment subscales: relationship with disordered eating/behaviours

Ten studies that used the IPPA as their attachment measure, explored the independent contributions of each subscale [61, 62, 64, 66, 67, 71, 74–76, 79].

#### Cross sectional

Brochu et al. investigated ED severity in adolescent females receiving specialised ED treatment [61]. Trust and good communication with peers were associated with reduced ED symptom severity. Only peer alienation contributed significantly to the prediction of ED severity, whereby the more alienation experienced, the greater the severity of symptoms. However, this relationship became non-significant when adjusted for low self-esteem and negative mood. Insecure attachment was associated with low self-esteem and negative mood, which were in turn significantly associated with ED symptom severity. External validity was limited by the female-only clinical population that neglects those with an ED but are not receiving treatment.

Cunha et al. and Lukas et al. both explored how IPPA subscale scoring differed between a clinical and non-clinical population [62, 67]. Both studies found that patients with AN reported lower trust and communication, as well as higher alienation, which emerged as the primary variable distinguishing those with AN from the control group.

Pace et al. similarly demonstrated that lower communication and trust scores were associated with greater binge eating symptoms. However, reduced perceived alienation scores in this study were also found to be correlated with higher risk binge eating symptoms [71].

A thesis by Gibbs observed differences in peer attachment between participants classified into groups based on the DSM-III criteria for anorexia nervosa. The groups included those who met the study’s criteria for anorexia nervosa, those who met the criteria but had a weight between 90 and 110% of the projected normal weight for height, those who met the criteria but also reported binge-eating behaviours, and those who did not meet the criteria for these groups [64]. Participants who reported behaviours related to anorexia and bulimia nervosa perceived greater alienation, which was the only subscale that distinguished between groups. However, the group labelled “bulimic” was created to account for the insufficient number of adolescents meeting the criteria for anorexia nervosa. Additionally, the DSM criteria used for group classification was modified to accommodate self-report methods and to improve sample size. This included changing the weight criteria for anorexia nervosa from > 25% weight loss to > 20% weight loss and using the Eating Attitudes Test to assess for body image disturbance, despite many of the questions relating to behaviours and attitudes with food rather than body image. The high risk of bias has resulted from the reliance on self-report methods to assess eating behaviours and infer diagnoses, as well as the modification of official criteria to increase sample size.

Laporta-Herrero et al. found that outpatients from an ED unit, compared to students who had never attended a mental health unit, had worse communication with friends and felt more alienated [66].

Sharpe et al. found that positive measures of trust and communication were not protective against eating pathology in non-clinical adolescent girls attending school [75]. Meanwhile, peer alienation was positively correlated with and predicted eating pathology, although it accounted for only 7% variance.

Schutz et al. found that only greater peer alienation was correlated with greater dietary restraint, extreme weight loss behaviours, and bulimic symptoms in non-clinical girls at secondary school [74]. Prevalence of perceived alienation was greatest in the top quartiles for dieting and bulimic symptoms.

#### Longitudinal

Burge et al. followed adolescents in school, measuring ED symptomology at baseline and 12 months later[76]. Baseline peer alienation was significantly correlated with follow-up ED symptomology. Alienation predicted 2% of the variance in follow-up symptomology, while trust and communication only did so in conjunction with initial symptomology as interaction terms. Although significant, the magnitude of predictions was small.

Thibault et al. conducted a longitudinal study in Canada to examine the impact of psychological, familial, and friendship factors on symptom severity in 143 adolescent girls, aged 12–17, admitted to specialised eating disorder treatment programmes[79]. The study utilised the IPPA to measure attachment at admission (T1) and the Eating Disorder Inventory‑3 (EDI‑3) to assess AN symptom severity after one year (T2). There were significant associations between greater AN symptom severity at T2 and poorer social functioning with peers, specifically greater alienation and lower communication and trust. Regression analysis showed that greater peer alienation at T1 was a significant predictor of AN symptom severity at T2. Mediation analysis revealed that while general psychological maladjustment at T1 remained a significant predictor of AN symptom severity at T2, peer alienation at T1 partially mediated this relationship. This indicates that peer alienation may contribute to the severity of AN symptoms by exacerbating the effects of psychological maladjustment.

### Peer attachment subscales: relationship with body dissatisfaction

Laporta-Herrero et al. studied a clinical group of outpatients for mental health services and a non-clinical group who had never attended a mental health unit [66]. They found that greater peer alienation was associated with body dissatisfaction, but only in those with a diagnosed ED. Selection of participants by psychologists is likely to have contributed to selection bias. Generalisability is hindered by exclusion of comorbidity.

Schutz et al. found a correlation between peer alienation and body dissatisfaction, after controlling for BMI and depression [74]. Those in the highest dissatisfaction quartile reported greater alienation from peers compared to the lowest quartile. Sharpe et al. also found that higher alienation was associated with body dissatisfaction and observed that dissatisfaction was associated with lower trust and communication scores [75].

## Discussion

This review aimed to explore the relationship between peer attachment and disordered eating behaviours or body dissatisfaction in adolescence. Most supported the presence of a relationship, although the nature of association varied. Three out of nineteen studies did not find any significant associations. There was strong evidence for the impact of perceived alienation from peers on ED outcomes, in comparison to the two positive subscales of trust and communication.

In this review, seven studies demonstrated that as overall security of attachment decreased, the likelihood of having an ED, being dissatisfied with the body, or having severe ED symptomology increased [62, 65, 67, 69, 71–73]. There is consistent evidence from previous research linking insecure attachment styles with parents or caregivers to a higher prevalence of dysregulated eating behaviours [39, 41, 90–92]; this review extends those findings to suggest that insecure attachment to peers may also be associated with similar outcomes. There may also be similarities in the mechanisms behind both, such as the nuances of different attachment styles. Gander et al. summarised how those with anxious attachment may be prone to negative affect and difficulties regulating emotions, which could tend towards binge-eating and purging behaviours [39]. Meanwhile individuals with avoidant attachment may suppress their emotions and lean towards dietary restriction. However, the strength of this conclusion is challenged by inconsistent results in regression analyses, where attachment items failed to significantly account for variances in ED pathology or body image.

While there is support for an association between insecure attachment and eating pathology, evidence that a *secure* attachment pattern could exert a protective effect is much weaker. Indeed, two high-quality longitudinal studies by Cortés-García et al. and Le Grange et al. demonstrated that higher peer attachment scores, reflecting attachment security, were associated with a higher risk for disordered eating [77, 78]. While attachment measures can provide insight into the quality of friendship, there is no indication of the attitudes and behaviours of the referenced security figure, at least not in the studies in this review. Greater attachment security is characterised by greater trust and communication but the nature of the beliefs or practices of peers may not be indicated. As trust develops in friends, so does acceptance of their attitudes and opinions into internal models [39–41]. Those with stronger attachment to friends may hold greater value in their friends’ beliefs about body weight and the social norm; this increases likelihood of internalisation of beliefs, criticisms and ideals, therefore perpetuating body dissatisfaction. This aligns with the results from Hold & Espelage and Hodges et al. who demonstrated that friendships that are highly supportive or characterised by companionship can contribute to amplified adverse outcomes [93, 94]. Agnew also found that those who were closely attached to “delinquent adolescents” would follow their behaviour [95].

Studies that observed individuals who had been diagnosed with an eating disorder did not demonstrate this variance in results [61, 62, 66, 67, 79]. These studies found similar results, showing lower attachment scores in individuals with an eating disorder diagnosis or with greater symptom severity, with alienation being a more prominent factor compared to trust and communication.

The protective role of attachment is also challenged by the consistent support for the comparative impact of perceived alienation on eating behaviours, where positive factors of trust and communication had little to no impact [66, 74–76]. The alienation subscale (IPPA) was often the only scale to have a significant correlation with ED symptomology or body dissatisfaction. This resonates with the conclusions of Domine et al., who found no significant association between disordered eating behaviour and peer relationships. This study solely utilised the absence of ‘positive’ features of friendship, such as trust, as the markers of poor relationship quality, but did not explore the presence of ‘negative’ features, such as isolation. While Domine et al. provides valuable insight to an under-represented group within this review by exclusively studying boys, omission of these ‘negative’ elements is not supported by our review, which demonstrated alienation’s significance compared to positive factors.

All studies with a body image outcome demonstrated that attachment insecurity to peers was associated with negative self-image [66, 69, 74, 75]. Body dissatisfaction and reduced attachment may reflect a general negative outlook, with these individuals perceiving their own bodies and friendships as having fewer positive features. This corresponds with what is known as the ‘general vulnerability view’ where insecurely attached individuals have a bias towards negative affect, such as discontent with their appearance and pessimism in judging friendships [96, 97]. However, it is important to note that the relationship between social interaction and eating disorders is likely to be bi-directional, where suffering from eating disorder symptoms could lead to withdrawal from friends and social isolation [23]. This could introduce a cycle of social isolation that promotes disordered eating and further fractures friendships.

The relationship between friends differs from familial relations in the way that friends can be chosen [98]. Peer attachment can encompass relationships with multiple friends and different friends may fulfil varying roles without reliance in a sole individual [99]. This contrasts with the dyadic relationship originally described in attachment theory [35]. However, friendships may be able to compensate for a lack of attachment with parents [48]. As one faces crisis, attachment theory dictates that they will seek their security figure, also known as the safe-haven function. Adolescents seek security, just as infants do, but the source of which is no longer limited to parents. When this figure is not perceived to be available, this may (a) perpetuate any beliefs of self-unimportance, and (b) drive the individual to resort to other coping methods, such as those involving food [100–102]. For some, eating may serve as a coping strategy in the face of distress, while for others the restriction of diet is used to prevent negative emotions [92]. Deaver et al. found that to negate negative affect, for example due to rejection from friends, some use binge-eating for temporary relief [103]. Thus, we can see that the fundamental functions of attachment remain but may change in nature. Rather than viewing attachment to parent and peer as independent entities, it may be of benefit to understand how the two interact in tandem [48, 104–107].

One study was able to also observe the role of attachment to siblings [70]. It can be argued that a sibling can be the first friend one acquires, and an early intimate relationship outside of that with a parent or carer. The influence of a sibling is both an important and unpredictable matter. This can range from the reinforcement of aggressive behaviour, to contributing to parental behaviour and knowledge [108, 109]. Although Pace et al. did not find the relationship between sibling attachment and disordered eating to be significant, the sparsity of literature on these matters should be noted. Siblings have been shown to have an impact on body dissatisfaction and eating behaviours and exploring this link may contribute further to this discussion [110, 111].

### Strengths and limitations

This review has strength in its novelty. To the authors’ knowledge, this is the first review that centres on attachment exclusively to peers. Secondly the content is aligned with the current direction of mental health research which is moving towards better understanding the role of interpersonal relationships in onset, maintenance, treatment, and recovery. This review could serve as an introduction to this concept, from which more research can be conducted into the clinical implications of attachment. Utilisation of the Inventory of Parent and Peer Attachment (IPPA) questionnaire is supported by its validity and wide usage [83, 112]. Additionally, most studies had a low risk of bias, largely due to the utilisation of validated attachment and outcome questionnaires.

However, the findings of this review should be approached with caution. Firstly, included studies were all completed in “Westernised” countries and so, along with the exclusion of non-English studies, generalisability to other countries or cultures is questionable. Additionally, three included studies were theses and were therefore not peer reviewed. Paucity of research in this field of interest is reflected in the 497 papers obtained from the initial search; however, this has contributed to this review’s strength in its broad search strategy.

Cross-sectional designs limit our ability to determine causality, as well as the direction of relationship between attachment and disordered eating. Given that only three studies use a longitudinal design and one of them is at high risk of bias, future research should aim to conduct more longitudinal studies.

Heterogeneity of outcome measures prevents comparison between studies. For example, some eating disorder questionnaires measured incidence of symptoms in the past 4 weeks, while others only ascertained overall or regularity of symptoms. Many studies recruited participants by advertising to parents. This biases the samples towards children whose parents have knowledge of mental health. Additionally, recruitment and selection through school neglects those who do not attend school, who are an arguably high-risk group for mental illness [113, 114]. Two studies exclude comorbidity which removes an important risk group due to the known links between depression or anxiety and eating disorders [39, 75, 88, 115].

Although validated, use of the IPPA as the attachment measure held multiple limitations. Firstly, interpretation of the score varied from classification of secure or insecure attachment to utilisation as a continuous scale. This does not align with the known styles of attachment, such as anxious and avoidant. Secondly, the self-report questionnaire risks recall bias. Although this tends towards subjectivity, it is likely that perceived attachment is more pertinent than observed behaviour. It is how one feels about relationships that represents the internal working model. Indeed, Iannotti & Bush argued that perceptions of friends’ behaviour, rather than actual behaviour, has stronger correlations with adolescent outcomes [116]. IPPA subscales may not reflect total peer attachment. The relationship of global IPPA scores, as per the original intention, and eating pathology in these studies cannot be concluded.

A common limitation across the studies was the failure to account for potential confounding variables, such as age and BMI [105, 117, 118]. Interestingly, some studies may have over-adjusted for certain factors. For instance, depression and anxiety were often controlled for, despite evidence suggesting they may act as mediators in the relationship between attachment and eating disorders [61, 72, 74, 75]. Adjusting for these variables could lead to an underestimation of the true associations, as it removes variance that may form part of the causal pathway. It is also important to consider that the link between insecure attachment and eating disorders may reflect a broader vulnerability to psychopathology. Insecure attachment has been associated with various psychological difficulties in adolescence, including depression and anxiety, which themselves are linked to eating disorders [88, 115]. This raises the possibility that the observed associations are not specific to eating pathology, but rather reflect a general susceptibility to mental health problems associated with insecure attachment.

Out of the seven single-sex samples in the review, only two observed boys [63, 65]. While it can be argued that girls represent the majority of the clinical eating disorder population, disordered eating behaviours remain a hidden issue in boys [119–121]. This reported imbalance in gender representation may not be due to true lower prevalence in boys, but the difficulty and differences in diagnosis for them [122]. An interesting avenue would be gender differences for these outcomes. Indeed, there are differences between genders for both influence from peers and attachment [106, 123, 124]. Girls seem to perceive greater attachment with friends compared to boys. Miljkovitch et al. found that attachment to peers compared to parents was greater in girls, with the opposite true for boys [104]. This should encourage greater exploration of these parameters in boys, who otherwise suffer underrepresentation.

### Implications

Insecure peer attachment may be a risk factor for disordered attitudes and behaviours. There is potential in developing interventions to improve these relationships to potentially reduce the risk of eating disorders in those with insecure attachment. The suggestions that secure peer attachment could worsen symptomology emphasises the need for a holistic approach. There should be a parallel effort to encourage positive attitudes towards bodies and healthy eating behaviours. Schools should work to promote values that centre in acceptance to prevent the perpetuation of harmful ideals. As for substance-abuse and sexual-health education, incorporating education in body weight and eating behaviours has been suggested [33]. The promotion of stronger relationships with friends, perhaps through peer-based education, could also be of benefit[22]. Some have even suggested intervention is best focussed in early or pre-adolescence [15]

An interesting direction for research is in peer attachment as a prognostic indicator, or the development of peer-related methods for psychotherapeutic processes. Those with avoidant attachment patterns were more likely to drop out of day treatments for anorexia nervosa [125, 126]. Thus, there may be benefit in utilising attachment relations in treatment. For example, involving peers in treatment programmes like group-counselling shows promise [28]. Others have shown that recruitment into these programmes with friends can improve treatment session attendance [127].

### Directions for future research

Longitudinal observation is of necessity to determining the relevance and potential of attachment security. It would be of great interest to measure peer attachment in early adolescence and follow the development of disordered eating behaviours into late adolescence. With consideration for the contrary finding that secure attachment increases symptom severity, it may be of benefit to simultaneously examine peer attachment and the peer’s opinions and perspectives on body image.

This review observed all ages of adolescence in unity. The impact of friendships has been shown to change with age, with some finding that the greatest influences from friends are seen in early adolescence [21, 105, 128]. However, evidence for this is scarce, with many unable to find a difference between ages [106]. Future studies should look to ascertain the progressions in peer attachment as one grows older.

Further avenues include the exploration of modifiers in the relationship between peer attachment and eating disorders. In this review, low self-esteem and depression were implicated as key players in indirect pathways between attachment and eating [61, 72, 74, 75]. Le Grange et al. alluded to mentalisation, reflecting recent interest in the interplay between mentalising capacity, attachment, and disordered eating [55, 78].

While many attachment measures exist for children and adults, they neglect isolating attachment to friends [59, 129]. Future research should look to utilise or develop other peer attachment measures, specifically those that measure relationships to peers independent to parents.

## Conclusion

Considering the well-established roles of attachment patterns and interpersonal relations in disordered eating, peer attachment is an area of significance to this field. It appears that insecure peer attachment may be a risk factor for disordered eating, but the protective potential of peer attachment remains unclear. More longitudinal studies that could disentangle the direction of relationship between peer attachment and eating disorders are required, as well as exploration into potential mediators. Finally, investigations into interventions that improve peer attachment are required to determine its clinical potential.

## Data Availability

No datasets were generated or analysed during the current study.

## Appendix 1: Search strategy

Search strategies used for electronic databases MEDLINE, Embase, PsychINFO, and Scopus.

## MEDLINE: 23.^rd^ April 2024

1. exp Adolescent/or Adolescen*.mp.

2. Youth*.mp.

3. Teen*.mp.

4. exp Adolescent Behavior/

5. exp Adolescent Psychiatry/or exp Adolescent Health/or exp Psychology, Adolescent/

6. 1 or 2 or 3 or 4 or 5

7. exp"Feeding and Eating Disorders"/or eating disorder*.mp.

8. disordered eating.mp.

9. eating behavio?r*.mp.

10. exp Body Image/or body image*.mp.

11. (body adj2 satisf*).mp

12. (body adj2 dissatisf*).

13. body confiden*.mp.

14. (bing* adj2 eat*).mp.

15. (purg* adj2 eat*).mp.

16. anorexia nervosa.mp.

17. bulimia nervosa.mp.

18. (restrict* adj2 eat*).mp.

19. (avoidant restrictive food intake disorder or ARFID).mp.

20. diabulimia.mp.

21. (Food adj1 addict*).mp.

22. orthorexia nervosa.mp.

23. obes*.mp.

24. overweight.mp.

25. exp Overnutrition/

26. 7 or 8 or 9 or 10 or 11 or 12 or 13 or 14 or 15 or 16 or 17 or 18 or 19 or 20 or 21 or 22 or 23 or 24 or 25

27. peer*.mp. or exp Peer Group/

28. exp Friends/or friend*.mp.

29. sibling*.mp.

30. exp Peer Influence/

31. social support.mp. or exp Social Support/

32. 27 or 28 or 29 or 30 or 31

33. attach*.mp.

34. exp Object Attachment/

35. (Friends and Family interview).mp.

36. Adolescent Friendship attachment scale.mp.

37. Experiences in Close relationships.mp.

38. People in My Life.mp.

39. (Inventory of Parent and Peer Attachment).mp.

40. 33 or 34 or 35 or 36 or 37 or 38 or 39

41. 6 and 26 and 32 and 40

## Appendix 1: Search strategy contd.

### Embase: 23^rd^ April 2024

1. exp adolescence/or exp adolescent/or adolescen*.mp.

2. youth*.mp.

3. teen*.mp.

4. exp adolescent behavior/

5. exp child psychology/

6. exp child psychiatry/

7. 1 or 2 or 3 or 4 or 5 or 6

8. exp eating disorder/or eating disorder*.mp.

9. disordered eating.mp.

10. eating behaviour*.mp.

11. body image*.mp.

12. (body adj2 satisf*).mp.

13. body confiden*.mp.

14. (Body adj2 dissatisf*).mp.

15. (Bing* adj2 eat*).mp.

16. (purg* adj2 eat*).mp.

17. anorexia nervosa.mp.

18. bulimia nervosa.mp.

19. (restrict* adj2 eat*).mp.

20. avoidant restrictive food intake disorder.mp.

21. arfid.mp.

22. diabulimia.mp.

23. (food* adj1 addict*).mp.

24. orthorexia.mp.

25. obes*.mp.

26. overweight.mp.

27. exp body image/

28. exp obesity/

29. exp food intake/

30. exp underweight/

31. 8 or 9 or 10 or 11 or 12 or 13 or 14 or 15 or 16 or 17 or 18 or 19 or 20 or 21 or 22 or 23 or 24 or 25 or 26 or 27 or 28 or 29 or 30

32. exp peer group/or Peer*.mp.

33. exp friend/or friend*.mp.

34. social support.mp. or exp social support/

35. sibling*.mp.

36. 32 or 33 or 34 or 35

37. attach*.mp.

38. exp emotional attachment/

39. (Friends and Family interview).mp.

40. Adolescent Friendship attachment scale.mp.

41. Experiences in Close relationships.mp.

42. (Inventory of Parent and Peer Attachment).mp.

43. People in My Life.mp.

44. 37 or 38 or 39 or 40 or 41 or 42 or 43

45. 7 and 31 and 36 and 44.

## Appendix 1: Search strategy contd.

### PsychINFO: 23^rd^ April 2024

1. adolescen*.mp.

2. teen*.mp.

3. youth*.mp.

4. exp Adolescent Psychiatry/or exp Adolescent Behavior/or exp Adolescent Psychotherapy/or exp Adolescent Attitudes/or exp Adolescent Psychology/or exp Adolescent Psychopathology/or exp Adolescent Health/

5. 1 or 2 or 3 or 4

6. exp Body Image/or exp Eating Disorders/or exp Eating Behavior/or exp Binge Eating/or exp Eating Attitudes/or eating disorder*.mp.

7. disordered eating.mp.

8. eating behavio?r*.mp.

9. exp Body Image Disturbances/or exp Body Image/or body image*.mp.

10. (body adj2 satisf*).mp.

11. body confiden*.mp.

12. (body adj2 dissatisf*).mp.

13. (bing* adj2 eat*).mp.

14. (purg* adj2 eat*).mp.

15. anorexia nervosa.mp.

16. bulimia nervosa.mp.

17. arfid.mp.

18. Avoidant restrictive food intake disorder.mp.

19. diabulimia.mp.

20. orthorexia.mp.

21. (restrict* adj2 eat*).mp.

22. obes*.mp.

23. overweight.mp. [mp = title, abstract, heading word, table of contents, key concepts, original title, tests & measures, mesh word].

24. exp Overweight/

25. exp Food Addiction/

26. exp Dietary Restraint/

27. exp Food Intake/

28. 6 or 7 or 8 or 9 or 10 or 11 or 12 or 13 or 14 or 15 or 16 or 17 or 18 or 19 or 20 or 21 or 22 or 23 or 24 or 25 or 26 or 27

29. peer*.mp.

30. exp Peers/or exp Peer Relations/or exp Friendship/or exp Social Support/or friend*.mp.

31. social support.mp. or exp Social Support/

32. exp Social Influences/

33. exp Siblings/or sibling*.mp.

34. 29 or 30 or 31 or 32 or 33

35. exp Attachment Behavior/or attach*.mp.

36. (Friends and Family interview).mp.

37. Adolescent Friendship attachment scale.mp.

38. Experiences in Close relationships.mp.

39. (Inventory of Parent and Peer Attachment).mp.

40. People in My Life.mp.

41. 35 or 36 or 37 or 38 or 39 or 40

42. 5 and 28 and 34 and 41.

## Appendix 1: Search strategy contd.

### SCOPUS: 23^rd^ April 2024

(adolescen* OR youth* OR teen*).

AND

(("eating disorder") OR ("Disordered eating") OR ("eating behaviour") OR ("body image") OR (body W/1 satisf*) OR ("body confidence") OR (body W/2 dissatisf*) OR (bing* W/2 eat*) OR (purg* W/2 eat*) OR (restrict* W/2 eat*) OR (“anorexia nervosa”) OR (“bulimia nervosa”) OR ({Avoidant restrictive food intake disorder}) OR (arfid) OR (diabulimia) OR (food AND addiction*) OR (orthorexia) OR (pica) OR (rumination) OR (obes*) OR (overweight)).

AND

(peer* OR friend* OR sibling*).

AND

(attach* OR {Friends and Family interview} OR {Adolescent Friendship attachment scale (AFAS)} OR {Experiences in Close relationships (ECR-RS)} OR {Inventory of Parent and Peer Attachment (IPPA)} OR {People in My Life (PIML)}).
